# Calpain 8/nCL-2 and Calpain 9/nCL-4 Constitute an Active Protease Complex, G-Calpain, Involved in Gastric Mucosal Defense

**DOI:** 10.1371/journal.pgen.1001040

**Published:** 2010-07-29

**Authors:** Shoji Hata, Manabu Abe, Hidenori Suzuki, Fujiko Kitamura, Noriko Toyama-Sorimachi, Keiko Abe, Kenji Sakimura, Hiroyuki Sorimachi

**Affiliations:** 1 Calpain Project, The Tokyo Metropolitan Institute of Medical Science (Rinshoken), Tokyo, Japan; 2 Department of Cellular Neurobiology, Brain Research Institute, Niigata University, Niigata, Japan; 3 Laboratory of Electron Microscopy, The Tokyo Metropolitan Institute of Medical Science (Rinshoken), Tokyo, Japan; 4 Department of Gastroenterology, National Center for Global Health and Medicine, Tokyo, Japan; 5 Department of Agricultural and Life Sciences, The University of Tokyo, Tokyo, Japan; 6 Core Research for Evolutional Science and Technology (CREST), Japan Science and Technology Agency (JST), Kawaguchi, Japan; Stanford University School of Medicine, United States of America

## Abstract

Calpains constitute a superfamily of Ca^2+^-dependent cysteine proteases, indispensable for various cellular processes. Among the 15 mammalian calpains, calpain 8/nCL-2 and calpain 9/nCL-4 are predominantly expressed in the gastrointestinal tract and are restricted to the gastric surface mucus (pit) cells in the stomach. Possible functions reported for calpain 8 are in vesicle trafficking between ER and Golgi, and calpain 9 are implicated in suppressing tumorigenesis. These highlight that calpains 8 and 9 are regulated differently from each other and from conventional calpains and, thus, have potentially important, specific functions in the gastrointestinal tract. However, there is no direct evidence implicating calpain 8 or 9 in human disease, and their properties and physiological functions are currently unknown. To address their physiological roles, we analyzed mice with mutations in the genes for these calpains, *Capn8* and *Capn9*. *Capn8^−/−^* and *Capn9^−/−^* mice were fertile, and their gastric mucosae appeared normal. However, both mice were susceptible to gastric mucosal injury induced by ethanol administration. Moreover, the *Capn8^−/−^* stomach showed significant decreases in both calpains 9 and 8, and the same was true for *Capn9^−/−^*. Consistent with this finding, in the wild-type stomach, calpains 8 and 9 formed a complex we termed “G-calpain,” in which both were essential for activity. This is the first example of a “hybrid” calpain complex. To address the physiological relevance of the calpain 8 proteolytic activity, we generated calpain 8:C105S “knock-in” (*Capn8^CS/CS^*) mice, which expressed a proteolytically inactive, but structurally intact, calpain 8. Although, unlike the *Capn8^−/−^* stomach, that of the *Capn8^CS/CS^* mice expressed a stable and active calpain 9, the mice were susceptible to ethanol-induced gastric injury. These results provide the first evidence that both of the gastrointestinal-tract-specific calpains are essential for gastric mucosal defense, and they point to G-calpain as a potential target for gastropathies caused by external stresses.

## Author Summary

The continuous or improper ingestion of irritants, including alcohol, nonsteroidal anti-inflammatory drugs (NSAIDs), and *Helicobacter pylori*, often leads to serious gastropathies, affecting a wide range of people. A complex gastric defense system helps protect against these threats, for example by secreting mucus. Here we report that two gastrointestinal-tract-specific calpains, calpain 8/nCL-2 and calpain 9/nCL-4, are involved in the mucosal defense against stress-induced gastropathies. Calpains are Ca^2+^-dependent cytosolic proteases that are indispensable for various cellular processes. Improper calpain activities can result in death or serious disorders, such as muscular dystrophies and lissencephaly, although no role for calpains in gastrointestinal diseases has been reported. Here we show that mice with mutations in the genes for calpains 8 and 9 are susceptible to alcohol-induced gastric injury. Moreover, these calpains form a stable complex, in which both molecules are essential for activity. Thus, human calpains 8 and 9 may contribute to the stomach's susceptibility to stress caused by irritants such as alcohol. Indeed, some reported human single nucleotide polymorphisms (SNPs) in these calpains are predicted to compromise their proteolytic activity. Our mutant mice provide unique animal models for potential human gastropathies caused by such SNPs.

## Introduction

Calpain (Clan CA-C2, EC 3.4.22.17) is a family of intracellular Ca^2+^-regulated cysteine proteases found in almost all eukaryotic and some bacterial, and all the members of this family share homology in the protease domain [1], [2]. Calpains play indispensable roles in biological processes including the cell cycle, apoptosis, platelet aggregation, and myoblast fusion, through the limited proteolytic cleavage of diverse substrates. Improper calpain activity often causes death or serious disorders, such as muscular dystrophies or lissencephaly [3]–[8].

**Figure 1:**
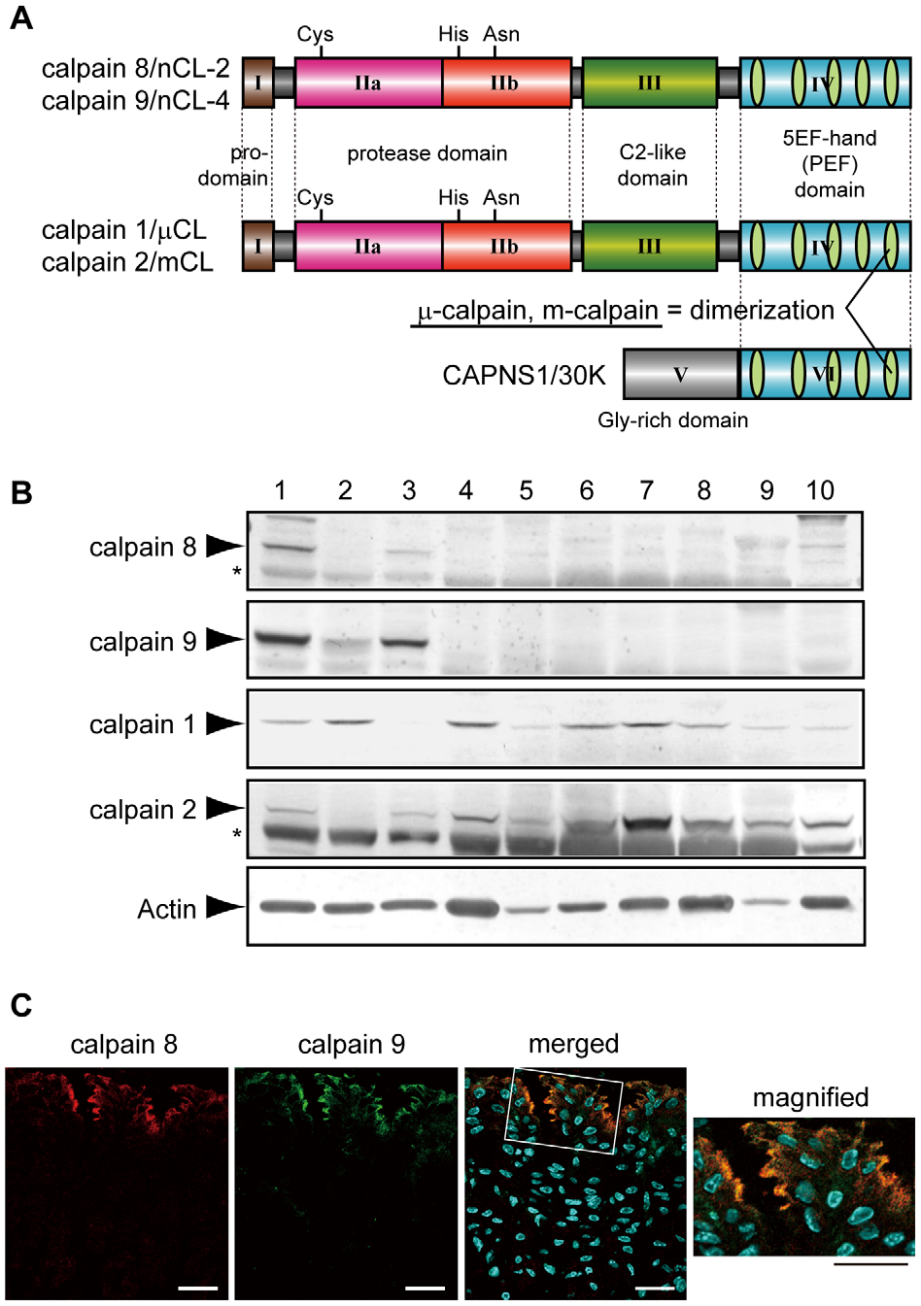
Calpains 8 and 9 showed the same tissue distribution and localization in the stomach. (A) Schematic illustrations of mammalian calpains 1, 2, 8, and 9. Calpains 8 and 9 have a typical domain structure like calpains 1 and 2: a regulatory N-terminal pro-domain (domain I), protease domain (subdomains IIa and IIb, also called subdomains I and II), C2-like Ca^2+^/phospholipid-binding domain (domain III), and penta-EF-hand (PEF) domain (domain IV). CAPNS1 includes a Glycine-rich domain (domain V) and PEF domain (domain VI). Calpain 8 shows high similarity to calpain 2 over the whole molecule (amino acid identities of the full-length protein and of domains II and IV are 61.5%, 73.4%, and 51.8%, respectively). Calpain 1 (or 2) forms a heterodimer with CAPNS1 to be μ-calpain (or m-calpain). (B) Western blot analysis of mouse tissues using antibodies against calpain 8, calpain 9, calpain 1, calpain 2, and β-actin. Twenty micrograms of tissue homogenate was used for each lane except for β-actin, for which 2 µg of homogenate was used. Lanes: 1, gastric mucosa; 2, jejunum; 3, colon; 4, spleen; 5, liver; 6, kidney; 7, lung; 8, uterus; 9, heart; 10, brain. Asterisks indicate non-specific signals originating from the secondary antibody. (C) Double-immunostaining of the mouse stomach was performed using antibodies against calpain 8 (red) and calpain 9 (green). The right panel is a magnified view of the boxed area. Blue signals represent DAPI-stained nuclei. Bars, 50 µm.

The mammalian calpains include both ubiquitous and tissue-specific members [2]. The gastrointestinal-tract (GI) expresses two major ubiquitous calpains, μ- and m-calpain, and two GI-specific ones, calpain 8/nCL-2 and calpain 9/nCL-4 [9], [10]. The μ- and m-calpains each form a heterodimer consisting of a distinct 80-kDa catalytic subunit (calpain 1/µCL and calpain 2/mCL, respectively) and a common 30-kDa regulatory subunit (CAPNS1/30K), which acts as a molecular chaperone for calpains 1 and 2. Calpains 8 and 9 have a typical domain structure like that of calpains 1 and 2: a regulatory N-terminal domain (domain I), protease domain (II, consisting of sub-domains IIa and IIb), C2-like Ca^2+^/phospholipid-binding domain (III), and penta-EF-hand (PEF) domain (IV) (Figure 1A).

Although calpains 8 and 9 share this domain structure with calpains 1 and 2, when expressed alone in cultured cells or *E. coli*, calpain 8 is active without CAPNS1 and forms a homo-oligomer [11], whereas calpain 9 requires the co-expression of CAPNS1 [12]. Possible functions for calpain 8 in vesicle trafficking between the ER and Golgi, and those for its homologue, XCL-2, in the embryogenesis of *Xenopus laevis* have been reported [13], [14]. Down-regulation of the calpain 9 transcript in a subset of gastric cancer patients and gastric cancer cell lines [15], and the association of a calpain 9 deficiency with the neoplastic transformation of naive cells have been reported [16], suggesting that calpain 9 plays a physiological role in the suppression of tumorigenesis.

These reports highlight that calpains 8 and 9 are regulated differently from each other and also from the conventional calpains, and thus have potentially important and specific functions in the GI that cannot be compensated for by μ- or m-calpain. However, calpains 8 and 9 have not been directly implicated in GI diseases, and their molecular properties and physiological functions have been elusive. To address these issues, we generated and analyzed mice with mutations in the genes for these calpains, *Capn8* and *Capn9*. Our results provide genetic evidence for the involvement of calpains 8 and 9 in gastric mucosal defense. Gastric mucosal integrity depends on a complex defense system that acts in the face of a variety of irritants, including alcohol, nonsteroidal anti-inflammatory drugs (NSAIDs), and *Helicobacter pylori*. The continuous or improper ingestion of these irritants often leads to serious gastropathies [17], [18]. Our results might therefore contribute to the development of new therapeutic strategies for human gastropathies induced by irritants.

## Results

### *Capn8^−/−^* and *Capn9^−/−^* mice are susceptible to ethanol-induced gastric mucosal injury

We found that calpains 8 and 9 were predominantly expressed in the stomach and, to a lesser extent, in the digestive tract (Figure 1B). The gastric epithelium is continuously stressed by low pH and digestive enzymes, and the pit cells play a central role in mucosal protection by secreting neutral mucus and bicarbonate. The pit cells originate from proliferative progenitor cells, which are located under the pits and differentiate during their migration to the pits. These cells undergo continuous turnover, being killed by apoptosis or necrosis within a few days, to achieve homeostasis of the gastric mucosa [19]. The localization of calpains 8 and 9 overlapped, and was predominant in the apical region of the non-proliferative middle and top pit cells, as previously reported [13] (Figure 1C).

**Figure 2:**
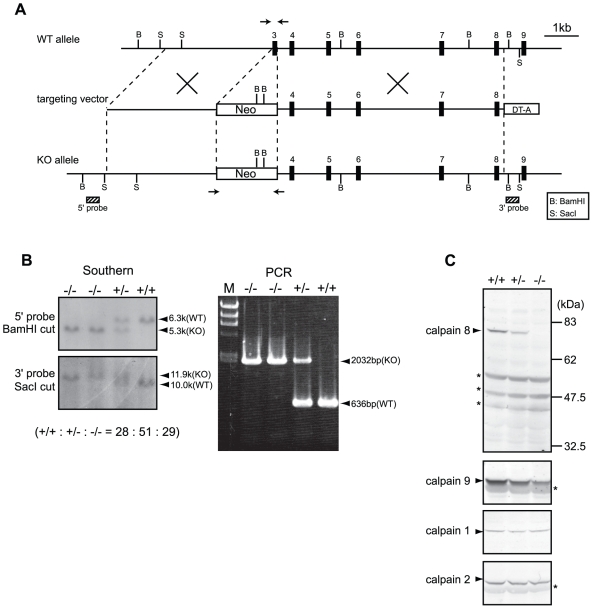
Generation of *Capn8^−/−^* mice. (A) Schematic representation of the targeting vector and WT and knock-out (KO) alleles of mouse *Capn8*. Exons 3 to 9 are indicated by black boxes with exon numbers. The probes for Southern blotting are shown as boxes with hatched lines. The PCR primer positions for genotyping are shown by arrows. Neo, neomycin-resistance gene; DT-A, diphtheria toxin A fragment. (B) Southern blot (left) and PCR (right) analyses of genomic DNA extracted from the tail of WT (+/+), *Capn8^+/−^* (+/−), and *Capn8^−/−^* (−/−) mice. The intercrossing of heterozygous mice generated WT, heterozygous, and homozygous mice at a ratio not significantly different from the expected Mendelian ratio. M, DNA marker. (C) Western blot analysis of gastric mucosal proteins prepared from WT (+/+), *Capn8* ^+/−^ (+/−), and *Capn8* ^−/−^ (−/−) littermate mice. Twenty micrograms of sample was used for each lane. Asterisks indicate non-specific signals.

**Figure 3:**
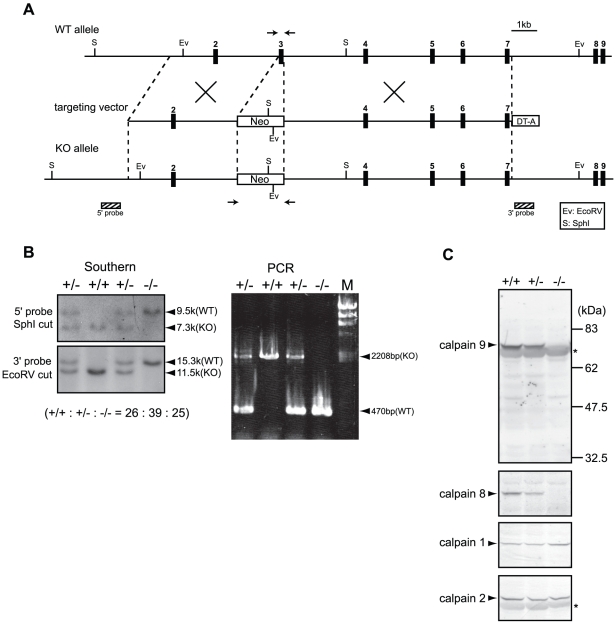
Generation of *Capn9^−/−^* mice. (A) Schematic representation of the targeting vector and WT and knock-out (KO) alleles of mouse *Capn9*. Exons 2 to 9 are indicated by black boxes with exon numbers. The probes for Southern blotting are shown as boxes with hatched lines. The PCR primer positions for genotyping are shown by arrows. Neo, neomycin-resistance gene; DT-A, diphtheria toxin A fragment. (B) Southern blot (left) and PCR (right) analyses of genomic DNA extracted from the tail of WT (+/+), *Capn9^+/−^* (+/−), and *Capn9^−/−^* (−/−) mice. Intercrossing of heterozygous mice generated WT, heterozygous, and homozygous mice at a ratio not significantly different from the expected Mendelian ratio. M, DNA marker. (C) Western blot analysis of gastric mucosal proteins prepared from WT (+/+), *Capn9* ^+/−^ (+/−), and *Capn9* ^−/−^ (−/−) mice. Twenty micrograms of sample was used for each lane. Asterisks indicate non-specific signals.

**Figure 4:**
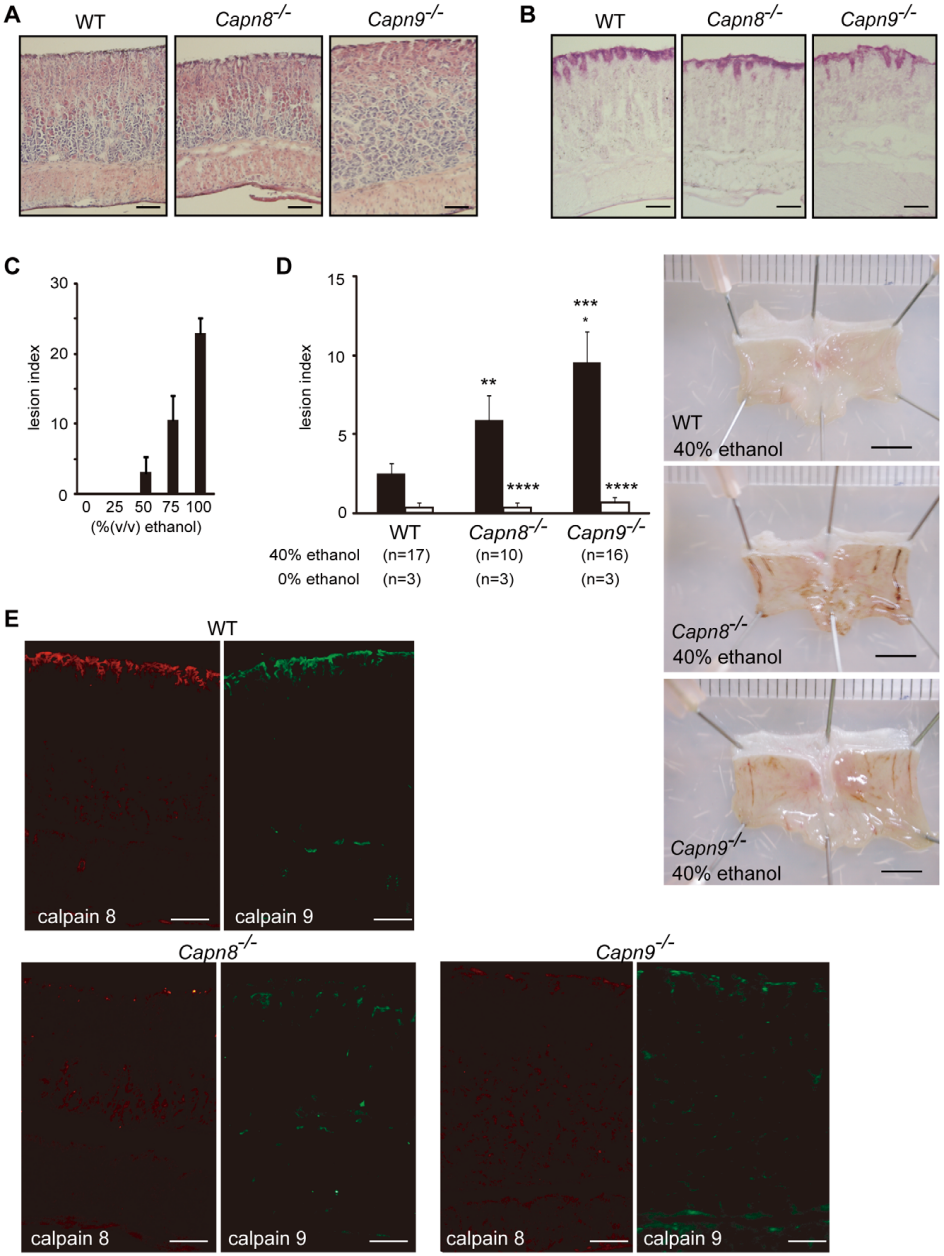
*Capn8* or *Capn9* deficiency increased the susceptibility to ethanol-induced gastric lesions. (A,B) The gastric mucosa of *Capn8* ^−/−^, *Capn9* ^−/−^, and WT mice was sectioned and analyzed by hematoxylin-eosin (H-E) staining (A), and periodic acid-Schiff (PAS) staining (B). Bars, 100 µm. (C) Susceptibility of WT mice to ethanol-induced gastric lesions. The indicated concentrations of ethanol were orally administered to WT mice, and the lesion index was determined (see Materials and Methods). Values are the means ± standard error (SEM). (n = 5). (D) (left) WT, *Capn8* ^−/−^, and *Capn9* ^−/−^ mice were orally given 0% (white bars) or 40% (black bars) ethanol, and the lesion indexes were determined (see Materials and Methods). Values are means ± SEM. *, *P*<0.01 vs. WT; **, *P*<0.05 vs. WT; Not significant vs. *Capn8* ^−/−^ mice (***), and vs. WT (****). (right) Representative macroscopic views of the gastric mucosa of each mouse 4 hours after 40% ethanol administration are shown. Bars, 5 mm. (E) The gastric mucosa of *Capn8* ^−/−^, *Capn9* ^−/−^, and WT mice was sectioned and analyzed by immunostaining using antibodies for calpain 8 (red) and calpain 9 (green). Bars, 100 µm.

To examine the functions of these proteins, we generated *Capn8^−/−^* and *Capn9^−/−^* mice (Figure 2A and 2B, Figure 3A and 3B), and confirmed the absence of their gene products by western blot analysis (Figure 2C, Figure 3C, upper panels). The *Capn8^−/−^* and *Capn9^−/−^* mice were fertile and showed no gross developmental abnormalities. Histological analysis of the stomach and intestines showed no obvious morphological abnormalities, such as mucosal hypertrophy or myxasthenia (Figure 4A and 4B for the stomach).

To evaluate the effects of the calpain 8 or 9 deficiency on stomach functions, we used an experimental gastric injury model. Ethanol has long been used to generate a hemorrhagic gastric mucosal injury in rodents [20]. Thus, we compared the development of gastric mucosal injury after the oral administration of ethanol among wild-type (WT), *Capn8^−/−^*, and *Capn9^−/−^* mice. The administration of 40% ethanol, which induced mild or little gastric mucosal injury in WT mice (Figure 4C), caused significantly enhanced gastric mucosal injuries in the *Capn8^−/−^* and *Capn9^−/−^* mice (Figure 4D). These mice showed no significant difference under the control condition (0% ethanol), indicating that calpains 8 and 9 are involved in protecting the gastric mucosa from ethanol-induced lesions.

**Figure 5:**
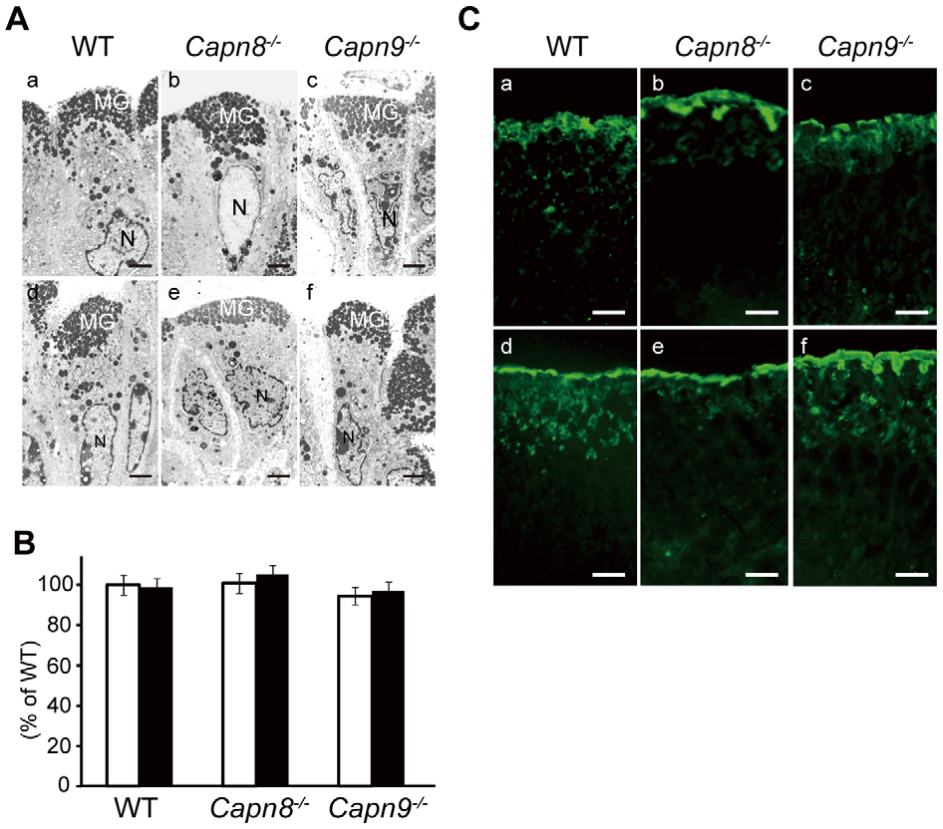
Effect of calpain 8 or 9 deficiency on mucous granule formation and secretion. (A) Electron micrographs of the pit cells of WT, *Capn8* ^−/−^, and *Capn9* ^−/−^ mice before (a–c) and after (d–f) ethanol administration. The gastric lumen is at the top of each photograph. MG, mucous granule; N, nuclei. Bars, 2 µm. (B) Comparison of the number of mucous granules per pit cell for WT, *Capn8* ^−/−^, and *Capn9* ^−/−^ mice before (white bars) and after (black bars) ethanol administration. Top-pit (fully differentiated) cells randomly selected from stomach samples were analyzed for each experimental group (n = 45, 41, and 44 for the WT, *Capn8* ^−/−^, and *Capn9* ^−/−^ mouse stomach groups before ethanol administration, and n = 36, 41, and 42 for those groups after the administration, respectively). The sum of the mucous granules divided by the number of cells analyzed was defined as the average number of granules per cell. Each mean value was standardized to that for the WT group before ethanol administration, which was defined as 100%. Values are means ± SEM. (C) Immunostaining of WT, *Capn8* ^−/−^, and *Capn9* ^−/−^ mouse stomachs before (a–c) and after (d–f) ethanol administration using an antibody for Muc5AC (green). Bars, 50 µm.

A complex network system functions to provide gastroprotection against gastric injury, and includes mucous production/secretion, and mucosal restitution by the pit cells [21]. To investigate the process in which calpains 8 and 9 are involved, we examined the effect of the calpain 8 or 9 deficiency on mucous granule production and secretion by electron microscopy and mucin immunostaining in the WT, *Capn8^−/−^*, and *Capn9^−/−^* mouse stomach. To monitor the early response in the pit cells, the stomachs were examined before and 1 hour after the administration of 40% ethanol. Consistent with the results shown in Figure 4B, there was no marked difference in granule formation among the WT, *Capn8^−/−^*, and *Capn9^−/−^* mouse stomachs before ethanol administration (Figure 5A, a–c). After the administration, these mouse stomachs showed neither a marked difference in appearance (Figure 5A, d–f) nor a significant difference in granule numbers (Figure 5B). The expression pattern of mucin-5AC (Muc5AC), a major component of the mucous secreted from the pit cells, did not differ markedly either (Figure 5C). These results indicated that calpains 8 and 9 do not function in granule production or secretion, and suggested that they are probably involved in the restitution of the mucosa by the pit cells. Further studies, however, are required to elucidate the function of calpains 8 and 9 in gastroprotection.

### Calpains 8 and 9 form a protease complex

**Figure 6:**
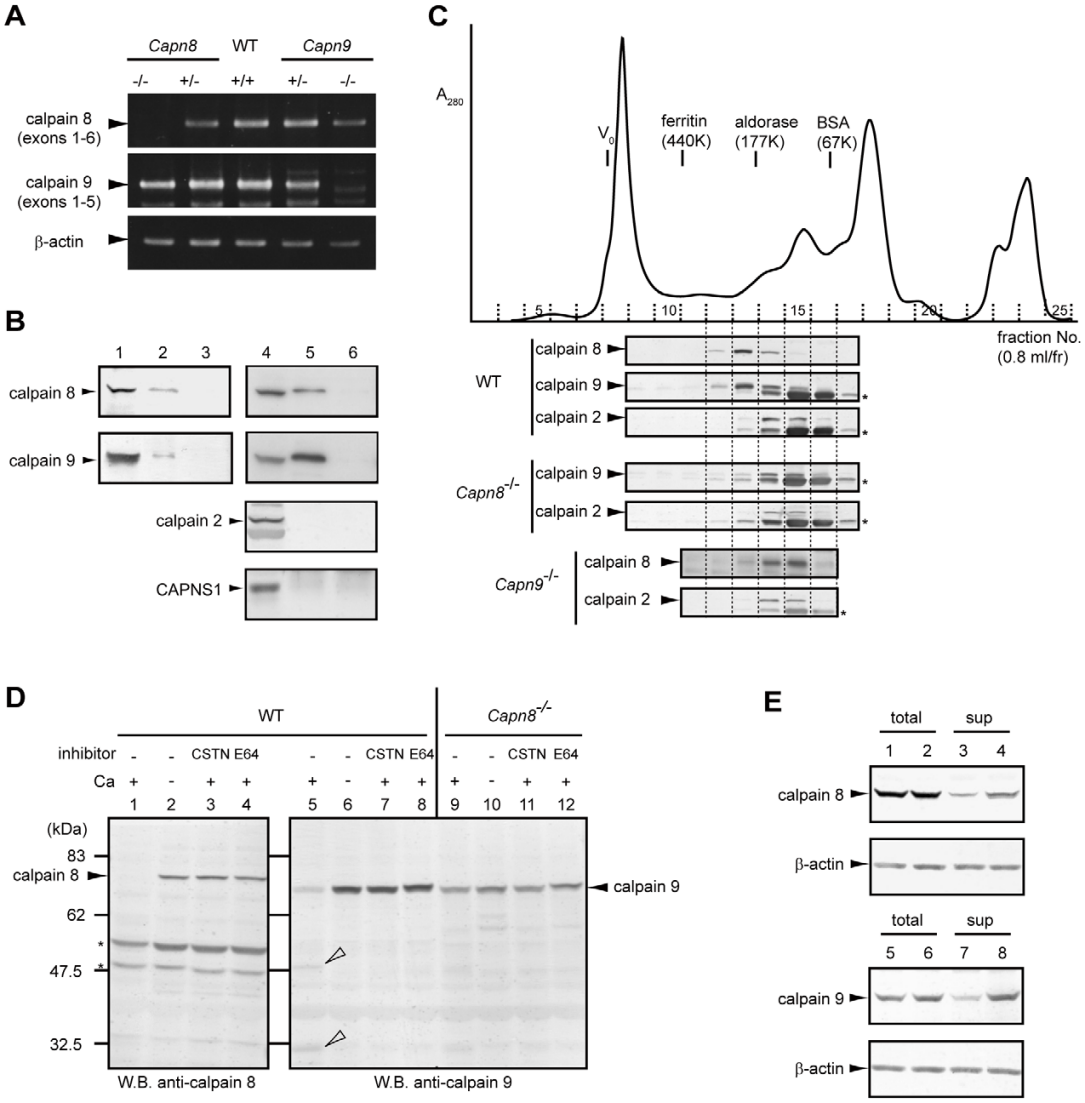
Calpains 8 and 9 form a complex. (A) RT-PCR analysis of calpains 8 and 9 mRNA in the WT, *Capn8* ^+/−^, *Capn8* ^−/−^, *Capn9* ^+/−^, and *Capn9* ^−/−^ gastric mucosa using primer pairs: calpain 8-5′/-3′ for calpain 8, calpain 9-5′/-3′ for calpain 9, and β-actin-5′/-3′ (see Table 2). (B) Gastric mucosal homogenate was immunoprecipitated without (lane 3) or with anti-calpain 8 (lane 2), anti-calpain 9 (lane 5), or absorbed-anti-calpain 9 (lane 6) antibodies, and subjected to western blotting using anti-calpain 8, anti-calpain 9, or anti-m-calpain (for calpain 2 and CAPNS1) antibodies. Lanes 1 and 4, 2% of the input used for immunoprecipitation. (C) Elution profile of the gastric mucosal proteins of WT, *Capn8* ^−/−^, and *Capn9* ^−/−^ mice from a Superdex 200 gel filtration column (solid line, A_280_). Fractions were subjected to western blotting using anti-calpain 8, anti-calpain 9, or anti-calpain 2 antibodies (lower panels). Asterisks indicate non-specific signals. (D) Gastric mucosal homogenates from WT (lanes 1–8) and *Capn8* ^−/−^ (lanes 9–12) mice were incubated with or without Ca^2+^ and inhibitors (CSTN, recombinant human calpastatin domain 1 fragment; E64, E64c). The samples were subjected to western blotting using anti-calpain 8 (lanes 1–4) or anti-calpain 9 (lanes 5–12) antibodies. Open arrowheads and asterisks indicate proteolytic fragments of calpain 9 and non-specific signals, respectively. (E) Mouse calpain 8 (lanes 1–4) was co-expressed with empty vector (lanes 1 and 3) or mouse calpain 9 (lanes 2 and 4) in COS7 cells, and mouse calpain 9 (lanes 5–8) was co-expressed with empty vector (lanes 5 and 7) or mouse calpain 8 (lanes 6 and 8). Soluble lysates (sup) were recovered by ultracentrifugation of the total lysates (total) of the treated COS7 cells. The same amounts of lysate were subjected to western blot analysis using anti-calpain 8 (lanes 1–4) or anti-calpain 9 (lanes 5–8) antibodies.

The effect of *Capn8^−/−^* and *Capn9^−/−^* on other calpain expressions was analyzed. Surprisingly, a significant decrease in calpain 9 accompanied the loss of calpain 8 in *Capn8^−/−^* mice, and similarly, calpain 8 was decreased in *Capn9^−/−^* mice; in contrast, calpains 1 and 2 were unaltered in the mice of any genotype (Figure 2C, Figure 3C). Consistent with these observations, immunohistochemistry also showed that calpains 9 and 8 were both downregulated in the stomachs of *Capn8^−/−^* or *Capn9^−/−^* mice (Figure 4E). Reverse transcribed-polymerase chain reaction (RT-PCR) analysis showed that the calpain 8 transcript in *Capn9^+/−^* and *Capn9^−/−^* mice and the calpain 9 transcript in *Capn8^+/−^* and *Capn8^−/−^* mice were comparable to their level in WT mice (Figure 6A), indicating that the observed down-regulation was at the protein level. Immunoprecipitation using lysate prepared from WT gastric mucosa co-precipitated calpains 8 and 9 (Figure 6B, lanes 2 and 5), whereas neither calpain 2 nor CAPNS1 was co-precipitated with calpain 9 (lane 5). These observations indicated that calpains 8 and 9 form a complex *in vivo*.

To investigate the nature of this complex, homogenate prepared from WT gastric mucosa was subjected to gel filtration column chromatography. Western blot analysis of the fractions revealed that calpains 8 and 9 were co-eluted with a single peak near the 177-kDa marker, whereas calpain 2 was eluted with a single peak between the 67- and 177-kDa markers, which corresponds to the molecular weight (MW) of m-calpain (calpain 2+CAPNS1, 110 kDa) (Figure 6C, WT). In contrast, in the homogenate from the *Capn8* ^−/−^ gastric mucosa, calpain 9, at much lower levels than in WT, showed an elution pattern similar to that of calpain 2 (“*Capn8^−/−^*”), as did calpain 8 in the *Capn9* ^−/−^ gastric mucosa (“*Capn9^−/−^*”), indicating that the residual calpain 9 does not form a homodimer without calpain 8, and *vice versa*.

### Calpains 8 and 9 are molecular chaperones for each other

Next, homogenate prepared from the *Capn8^−/−^* gastric mucosa was incubated with or without Ca^2+^. In WT mice, calpains 8 and 9 showed Ca^2+^-dependent autolysis (Figure 6D, lanes 1 and 5), which was inhibited by the addition of a recombinant calpastatin domain 1 fragment, one of four inhibitory units of an endogenous calpain-specific inhibitor protein, or of E64c, a more general cysteine protease inhibitor (lanes 3, 4, 7, and 8). As seen for calpain 2 in *Capns1^−/−^* mouse embryos, which are deficient in CAPNS1 [22], the amount of calpain 9 in the *Capn8^−/−^* mice was significantly decreased, as described above, and it lacked proteolytic activity, even in the presence of Ca^2+^ (lane 9). Consistent with this finding, in transfected COS7 cells, calpains 8 and 9 were more abundant in the supernatant fraction when both were co-expressed than when either molecule was expressed singly (Figure 6E, lanes 3, 4, 7, and 8). These results indicated that calpains 8 and 9 function as molecular chaperones for each other in the complex, like CAPNS1 does for calpain 2.

### Calpain 8 protease activity is essential for gastric mucosal defense

**Figure 7:**
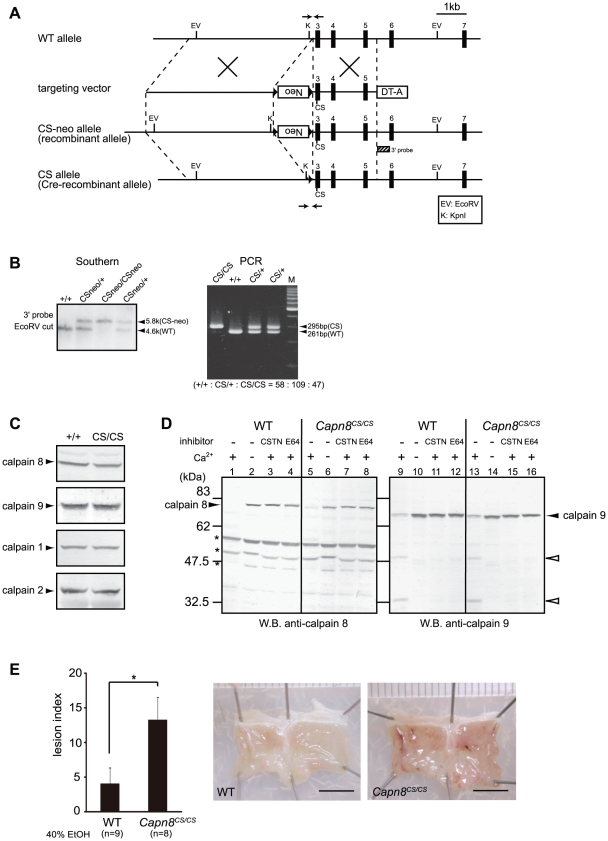
*Capn8^CS/CS^* mice are susceptible to ethanol-induced mucosal lesions. (A) Schematic representation of the targeting vector and WT, CS-neo, and CS alleles of mouse *Capn8*. Exons 3 to 7 are indicated by black boxes with exon numbers. The 3′-probe for Southern blotting is shown as a box with hatched lines. The PCR primer positions for the genotyping of Cre-recombinant mice are shown by arrows. Neo, neomycin-resistance gene; DT-A, diphtheria toxin A fragment. (B) (left) Southern blot analysis of genomic DNA extracted from the tail of WT, *Capn8^CSneo/+^* (CSneo/+), and *Capn8^CSneo/CSneo^* (CSneo/CSneo) mice. (right) PCR analysis of genomic DNA extracted from the tail of WT (+/+), *Capn8^CS/+^* (CS/+), and *Capn8^CS/CS^* (CS/CS) mice. Intercrossing of heterozygous mice generated wild-type, heterozygous, and homozygous mice at a ratio not significantly different from the expected Mendelian ratio. M, DNA marker. (C) Western blot analysis of the gastric mucosal homogenates (20 µg) prepared from WT and *Capn8^CS/CS^* (CS/CS) mice. (D) The gastric mucosal homogenates from WT (lanes 1–4 and 9–12) and *Capn8^CS/CS^* (lanes 5–8 and 13–16) mice were incubated with or without Ca^2+^ and inhibitors, as indicated (CSTN, recombinant human calpastatin domain 1 fragment; E64, E64c). The samples were subjected to western blot analysis using an anti-calpain 8 (lanes 1–8) or anti-calpain 9 (lanes 9–16) antibody. Open arrowheads and asterisks indicate proteolytic fragments of calpain 9 and non-specific signals, respectively. (E) (left) WT and *Capn8^CS/CS^* mice were orally given 40% ethanol, and the lesion index was determined. Values are the means ± SEM. *, *P*<0.05 vs. WT. (right) Representative macroscopic views of the gastric mucosa of WT and *Capn8^CS/CS^* mice 4 hours after ethanol administration. Bars, 5 mm.

Recently, calpain 6, a unique non-proteolytic member of the mammalian calpain family with a naturally inactive catalytic site, was reported to interact with microtubules and microfilaments and to be involved in their regulation [23]. Thus, calpain 6 is likely to function as a structural element. To examine whether the calpain 8/9 complex functions as a structural component or protease, we generated and analyzed calpain 8:C105S knock-in (*Capn8^CS/CS^*) mice, which express proteolytically inactive but structurally intact calpain 8 (Figure 7A and 7B). Like the *Capn8^−/−^* mice, *Capn8^CS/CS^* mice were fertile with no apparent abnormalities. Notably, the protein levels of calpains 8 and 9 as well as of calpains 1 and 2 were unaltered between the WT and *Capn8^CS/CS^* mice (Figure 6C). In the homogenate of the *Capn8^CS/CS^* gastric mucosa, calpain 9 showed Ca^2+^-dependent autolysis, which was inhibited by the calpastatin domain 1 fragment or E64c (Figure 7D, lanes 13–16). Notably, the calpain 8:C105S protein was almost completely proteolyzed Ca^2+^-dependently (Figure 7D, lane 5). This finding strongly suggests that, in the calpain 8/9 complex, not only intramolecular autolysis but also intermolecular proteolysis takes place, as in the activation of μ- and m-calpains, which involves the proteolysis of CAPNS1 [2]. At present, it cannot be completely excluded that μ- and/or m-calpain in the same fraction proteolyzed the calpain 8:C105S protein.

To our surprise, the oral administration of 40% ethanol significantly induced gastric injury in *Capn8^CS/CS^* mice (Figure 7E), with a severity comparable to that in *Capn8^−/−^* and *Capn9^−/−^* mice. This result indicated that the proteolytic activity of calpain 8 is essential for gastric mucosal defense, even though inactive calpain 8 forms a stable complex containing proteolytically active calpain 9.

## Discussion

The functions of tissue-specific calpains are most likely relevant to the tissues expressing them; therefore, there is an advantage to studying them over ubiquitous calpains, which are indispensable in embryogenesis or show functional redundancy [22], [24], [25]. For example, skeletal muscle-specific calpain 3/p94 is a product of the gene responsible for limb-girdle muscular dystrophy type 2A (LGMD2A) [4], demonstrating that the inactivation of tissue-specific calpains is a potential direct strategy for understanding some of the calpain functions. In this study, we investigated the gastrointestinal-tract-specific calpains by using gene targeting in mice, and obtained evidence that calpains 8 and 9 play a protective role in the gastric mucosa by forming a protease complex. This is the first report of the *in vivo* significance and properties of the gastrointestinal-tract-specific calpains.

The ethanol stress-dependent phenotype of the *Capn8^−/−^* and *Capn9^−/−^* mice and limited localization of calpains 8 and 9 suggest that these mutant mice have mild defects in their pit cells, and/or in their stress-induced immediate response for mucosal protection. So far, growth factors, heat shock proteins, and gastric mucosal blood flow have been reported to be important for gastric mucosal protection and restitution [26]–[28]. Notably, here we found that calpains 8 and 9 are also important for these functions, illuminating a novel research area for gastroenterology.

It is striking that calpains 8 and 9 form a complex in which both proteins are essential for stability and activity. This finding explains the co-localization of calpains 8 and 9 in the stomach, and why *Capn8^−/−^* and *Capn9^−/−^* mice showed the same phenotype without functional compensation between calpains 8 and 9. The MW of the complex (∼180 kDa) indicates that it contains at most one molecule each of calpain 8 and 9, although the possibility of one or more small additional subunits cannot be eliminated. It is curious that only calpains 8 and 9 form this complex, despite the highly similar overall amino acid sequences among calpain 1, calpain 2, calpain 8, and calpain 9. Recently, a comparison of the 3D structures of calpain 1, calpain 2, and calpain 9 revealed divergent mechanisms for their activation and regulation [29]. Calpains 8 and 9 probably evolved to have local structures specific for binding each other. We propose calling the enzyme entity composed of calpains 8 and 9 in the stomach, “Gastric calpain” (“G-calpain”), a novel “hybrid” type of calpain enzyme composed of different calpain catalytic subunits, which has never been reported for any kind of calpain.

As shown in Table 1, in the NCBI single nucleotide polymorphism (SNP) database, 3 and 15 missense polymorphisms in calpains 8 and 9 were found, respectively. Among these SNPs, the calpain 8:A136V, calpain 9:A102V, and calpain 9:R277W polymorphisms represent changes in amino acid residues that are highly conserved among calpain family members. Moreover, the amino acid positions of calpain 8:A136 and calpain 9:R277 correspond to calpain 3:A160 and calpain 3:R357, respectively, the sites of the reported LGMD2A pathogenic mutations, calpain 3:A160Q/P and calpain 3:R357W. These findings strongly suggest that some of these SNPs compromise the proteolytic activity of calpain 8 or calpain 9. Frame-shift SNPs are also expected to drastically change the nature of calpain 9 by truncating the C-terminal EF-hand motif(s). Therefore, it is tempting to speculate that these SNPs are related to a susceptibility to human gastropathies caused by irritants such as alcohol. It should be noted that R277W SNP shows a global frequency of 0.006, which means that one out of every 28,000 people probably has only inactive calpain 9 due to homozygous R277W alleles.

**Table 1:** Missense SNPs in human *CAPN8* and *CAPN9* and related LGMD2A pathogenic mutations in *CAPN3*.

calpain	SNPs*1	domain	Allele frequency (No. in parentheses: chromosome sample count)*2	Conser-vation*3	LGMD2A*4	Note
calpain 8 (NP_001137434)	A136V	IIa	N.D.	**11/14**	A160Q, A160P	
	S245Y	IIb	N.D.	3/14		
	Q386fs:PVQNPFG*	IIb	N.D.	-		
calpain 9 (NP_006606)	A102V	IIa	global (178): 0.017, European (120)African American (124), [unknown] (120): 0Sub-Saharan African (120), Asian (178): 0.078	**13/14**	Next to A133V	A133 of calpain 3 is also well conserved (11/14).
	S122R	IIa	global (180): 0.100, multiple (68): 0.074[unknown] (118): 0.102, European (118): 0.136African American (122): 0.172Sub-Saharan African (120): 0.233Asian (180): 0.067	8/14		
	D164N	IIa	global (180): 0.006	1/14		
	I234T	IIb	global (172): 0.006, multiple (76): 0.013	6/14		
	A239T	IIb	global (180): 0.006, multiple (78): 0.013	4/14		
	R277W	IIb	global (178): 0.006	**11/14**	R357W	
	K322Q	IIb	global (178): 0.270, multiple (78): 0.282[unknown] (52): 0.308, European (356): 0.267African American (258): 0.340Sub-Saharan African (356): 0.419Caucasian (92): 0.360, Asian (872): 0.177	1/14		
	H327Q	IIb	global (178): 0.034, multiple (78): 0.038Sub-Saharan African (120): 0.200European (120): 0, Asian (180): 0	5/14		
	E342K	IIb/III	global (180): 0.039, multiple (78): 0.064[unknown] (120): 0.017, European (408): 0African American (170): 0.088Sub-Saharan African (360): 0.158Asian (588): 0	2/14		
	R458W	III	global (166): 0.006	8/14		
	L468R	III	multiple (78): 0.013	3/14		
	R522W	IV	global (178): 0.197, multiple (72): 0.181[unknown] (118): 0.186European (408): 0.157, Asian (654): 0.072Sub-Saharan African (356): 0.250African American (166): 0.356	1/9		
	K553fs:RTSNSRS*	IV	N.D.	-		Deletion of 2nd to 5th EF-hand motifs
	M611I	IV	global (178): 0.056, multiple (76): 0.079[unknown] (120): 0.017, European (408): 0African American (170): 0.259Sub-Saharan African (360): 0.192Asian (588): 0	4/9		
	K673fs:GVHSSQYK*	IV	N.D.	-		Deletion of 5th EF-hand motif

*1: From the NCBI SNP database: http://www.ncbi.nlm.nih.gov/SNP/.

*2: Multiple data were combined to obtain the total ratio.

*3: Domains IIa and IIb of human CAPN1–3, 5–14, and SOLH, domain III of human CAPN1–3, 5–9,11–14, and CAPN10 (two domains), and domain IV of human CAPN1–3, 8–9, 11–14 were compared, and identical residues were counted.

*4: From the Leiden Muscular Dystrophy database: http://www.dmd.nl/capn3_home.html. The residue numbers correspond to that of calpain 3/p94 (NP_000061).

**Figure 8:**
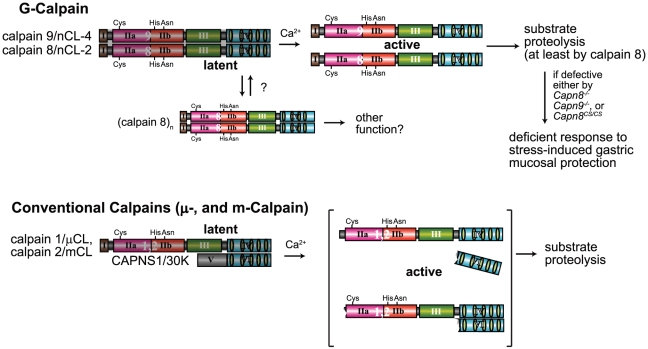
Hypothetical scheme for calpain activation in the stomach pit cells. (Upper) In the stomach pit cells, in addition to the conventional μ- and m-calpains, G-calpain is predominantly expressed. To be activated by Ca^2+^, G-calpain requires both catalytic subunits, which intramolecularly and intermolecularly autolyze, probably dissociating from each other. The proteolytic activity of at least calpain 8 is essential for the physiological function of G-calpain, that is, stress-induced gastric mucosal protection. We previously found that calpain 8, when transiently expressed without calpain 9 in cultured cells or in *E. coli*, forms homo-oligomers [11]. Although the physiological significance of this homo-oligomerization is unclear, it may play a role in modulating the activation of G-calpain under certain conditions. (Lower) In contrast, conventional μ- and m-calpains require a regulatory subunit, CAPNS1, but not other catalytic subunits, to be Ca^2+^-dependently activated to proteolyze their substrates, although it remains controversial whether or not their activation involves the dissociation of subunits, as illustrated here [40]–[42]. Since the stomach is under frequent stress, an extra calpain system in addition to the conventional one may have been required to respond swiftly to stresses.

What is the physiological significance of the calpain 8/9 complex formation? *Capn8^CS/CS^* mice, in which the calpain 9 protein level and proteolytic activity were unaltered, also showed a susceptibility to ethanol-induced gastric injury. This finding indicates that the activity of calpain 9 alone and inactive calpain 8 with its proteolyzed fragments were insufficient for specific functions of the gastric pit cells, and leads to a new model for calpain activation in the G-calpain system (Figure 8). In this model, increased intracellular Ca^2+^ induces the activation of calpains 8 and 9 at the same time and location, which is specifically required for pit-cell function, in addition to the conventional calpain system. Identification of the *in vivo* substrates for G-calpain is an important issue for future research. In this regard, one promising candidate is β-COP [30], [31], a subunit of the COP-I vesicle coatomer complex, which is involved in Golgi-endoplasmic reticulum membrane trafficking. We previously identified β-COP as an *in vitro* substrate for calpain 8 [13]. On the other hand, we also showed that G-calpain is not involved in mucus production or secretion (see Figure 5), which suggested that G-calpain functions in the mucosal restitution by pit cells after gastric injury. Consistent with this idea, conventional calpains are involved in cell migration [32]. Whether or not β-COP is relevant to this process is a very important issue to be elucidated in the future.

In humans, mutations of *CAPN3* were shown to be responsible for muscular dystrophy by forward genetics [4], and an intron polymorphism of *CAPN10* was reported to be associated with type 2 diabetes by statistical genetics [3]. This is the first report in which disease-responsible calpain genes were identified by reverse genetics. Our results also identify *CAPN8* and *CAPN9* as promising targets for various stress-induced human gastropathies. Our mutant mice described here represent potentially helpful tools for studying such diseases.

## Materials and Methods

### Experimental animals

All procedures using experimental animals were approved by the Experimental Animal Care and Use Committee of Rinshoken, and the animals were treated according to the committee's guidelines. C57BL/6 and ICR mice were purchased from Nihon CLEA. EIIa-Cre transgenic mice [33] were purchased from the Jackson Laboratory.

### Homologous recombination of embryonic stem (ES) cells

Mouse BAC clones containing *Capn8* or *Capn9* were purchased from Children's Hospital Oakland Research Institute. The genomic fragments used were amplified by PCR using Phusion high fidelity DNA polymerase (Finnzymes). Targeting vectors for *Capn8^−/−^* and *Capn9^−/−^* mice were constructed by replacing exon 3, which includes the codon for the active-site residue Cys, with a neomycin-resistance gene (neo) cassette (Figure 2A, Figure 3A). For the calpain 8:C105S knock-in (*Capn8^CS/CS^*) mice, Cys105 of calpain 8 was mutated to serine (Cys105Ser). A neo cassette flanked by loxP was inserted at intron 2 (Figure 7A). For the *Capn8^−/−^* mice, a 3.2-kbp fragment containing intron 2, and a 6.8-kbp fragment containing exons 4 to 8 were amplified and used as the 5′-arm and 3′-arm, respectively. For the *Capn9^−/−^* mice, a 4.2-kbp fragment containing exon 2, and an 8.6-kbp fragment containing exons 4 to 7 were used. For the *Capn8^CS/CS^* mice, a 4.3-kbp fragment containing intron 2, and a 2.1-kbp fragment containing exons 3 to 5 were used. For negative selection, a diphtheria toxin A fragment with an MC1 promoter [34] was attached downstream of the 3′ arms. The targeting vectors for *Capn8^−/−^* and *Capn9^−/−^* mice were linearized using SalI and electroporated into the C57BL/6N-derived ES cell line, RENKA [35]. For the *Capn8^CS/CS^* mice, the targeting vector was linearized using NotI and electroporated into TT2 ES cells [36]. The G418-resistant clones were screened, and the correctly targeted clones were confirmed by Southern blotting (Figure 2B, Figure 3B, Figure 7B). For Southern blotting, genomic DNA was digested with the appropriate restriction enzymes, and hybridized with the ^32^P-labeled probes shown in Figure 2A, Figure 3A, and Figure 7A.

### Generation of *Capn8^−/−^*, *Capn9^−/−^*, and *Capn8^CS/CS^* mice

The generation of chimeric males was achieved by the aggregation method for the *Capn8^−/−^* and *Capn9^−/−^* mice, and by the injection method for the *Capn8^CS/CS^* knock-in mice [36], [37]. Briefly, 8-cell-stage embryos were flushed out of the fallopian tubes of ICR females. In the aggregation method, the embryos were treated with acid Tyrode's buffer (Sigma) to remove the zona pellucida, and each embryo was placed in a depression created by an aggregation needle (Biological Laboratory Equipment) in a 35-mm-diameter culture dish containing M16 medium (Sigma). Five to ten positive ES cells were then placed adjacent to each embryo in the depression. In the injection method, five to ten positive ES cells were injected into each 8-cell-stage embryo. After an overnight culture, the ES cell-containing embryos that developed to the blastocyst stage were transplanted into the uterus of pseudo-pregnant ICR females. Germline transmitted chimeric males were crossed with C57BL/6N females for the *Capn8^−/−^* and *Capn9^−/−^* mice, and with C57BL/6J females for the calpain 8 CS knock-in mice, to generate heterozygotes. For the *Capn8^CS/CS^* mice, the heterozygotes were crossed with EIIa-Cre transgenic mice, in which Cre recombinase is expressed from the zygote stage, to remove the neo cassette from intron 2. The resultant mice were then backcrossed with C57BL/6J mice for 10 generations. The PCR primers used for genotyping are listed in Table 2. The mice were housed in pathogen-free facilities at our institute.

**Table 2:** PCR primers used in this study.

Capn8KO-5′	5′-gcctaagctgaatgtgtgtgcacttc-3′
Capn8KO-3′	5′-aacttggctgcgcttccagagtttct-3′
Capn9KO-5′	5′-cagggatccctgtgtctgaaaccagtc-3′
Capn9KO-3′	5′-ggaagatacccacatggtgtgacacca-3′
Capn8CS-5′	5′-taggagagtgctgtggtacctttggtcgac-3′
Capn8CS-3′	5′-gaattcctgcgacagggttatgtataaaca-3′
Calpain 8-5′	5′-tacagggatcttggaccaggctctgca-3′
Calpain 8-3′	5′-cttctgcttcggctgcgtttgaga-3′
Calpain 9-5′	5′-atgccttacctgcatcggtcc-3′
Calpain 9-3′	5′-ggatttggaagttctcagccacac-3′
β-actin-5′	5′-caggagatggccactgccgca-3′
β-actin-3′	5′-tccttctgcatcctgtcagca-3′

### Antibodies and reagents

The anti-calpain 8 domain III polyclonal antibody (ab28215), anti-calpain 9 monoclonal antibody (3A6), anti-calpain 9 polyclonal antibody (V-18), anti-Muc5AC monoclonal antibody (clone 45M1), and anti-β-actin monoclonal antibody (clone AC-15) were purchased from Abcam, Abnova, Santa Cruz, Thermo Scientific, and Sigma, respectively. The anti-calpain 2 monoclonal antibody (4B9F1), anti-m-calpain polyclonal antibody, and anti-calpain 1 polyclonal antibody were kind gifts from Dr. Seiichi Kawashima, Dr. Hideo Sugita, and Dr. Gen Kudo, respectively. Protease inhibitors (a recombinant human calpastatin domain 1 fragment (TaKaRa, No. 7316), E64c, PMSF, and pepstatin A) and chemical reagents were purchased from TaKaRa, Peptide Institute, Sigma, and Kanto Chemical.

### Histological analysis

Stomach and intestine samples were fixed and immunostained as described previously [13], and the signals were detected by LSM510 META confocal microscopy (Zeiss). For H-E staining, the sections were stained with Mayer's hematoxylin, followed by eosin staining. For PAS staining, the sections were pre-treated with 0.5% periodic acid and stained with Schiff's reagent (Polysciences). H-E- and PAS-stained sections were mounted with Mount-Quick (Daido Sangyo), and viewed with a BX60 microscope (Olympus).

The gastric ulcerogenic response was examined as described previously, with some modifications [38], [39]. Mice were fasted for 20 hours before the experiment, but were allowed free access to water. The mice were given 5 ml/kg body weight of 40% ethanol or water (0% ethanol) orally. After 4 hours, the mice were sacrificed by cervical dislocation. The stomachs were removed, opened along the grate curvature, and rinsed gently in PBS for the macroscopic evaluation of gastric lesions. The severity of gastric lesions was scored according to the following lesion index: 0 = no lesion; 1 = punctuate lesion; 2 = short linear ulcer (less than 2 mm); 3 = medium linear ulcer (2–4 mm); 4 = long linear ulcer (more than 4 mm). The sum of the total scores divided by the number of samples was defined as the mean lesion index.

To evaluate the involvement of calpains 8 and 9 in mucous formation and secretion, six experimental groups were prepared, corresponding to WT, *Capn8^−/−^*, and *Capn9^−/−^* mice before or after 40% ethanol administration. Two mice were used for each experimental group. The mice were sacrificed 1 hour after ethanol administration, and stomach samples were prepared as described above, and subjected to electron microscopy and immunostaining.

For electron microscopy, the stomach samples were fixed with 0.1 M sodium phosphate buffer (pH 7.4) containing 4% paraformaldehyde and 1% glutaraldehyde. After a 2-hour fixation at 4°C, the samples were washed with 0.1 M sodium phosphate buffer (pH 7.4), post-fixed in the same buffer containing 1% osmium tetroxide for 60 min, dehydrated with a graded ethanol series, and embedded in Epon 812 (Shell Chemicals). Semi-thin sections stained with toluidine blue were prepared, to verify the orientation of the sections by light microscopy. Ultra-thin sections were then prepared and mounted on copper grids, electron-stained with uranyl acetate and lead citrate, and examined with a JEM 1230 electron microscope (Japan Electron Optics Laboratory) at an accelerating voltage of 80 kV.

### Biochemical analyses

Mouse gastric mucosa was scraped from the underlying muscularis layer, homogenized in the appropriate buffer, and subjected to immunoprecipitation, incubation assay, or gel-filtration analyses. For immunoprecipitation, the gastric mucosal homogenate from a WT, *Capn8* ^−/−^, or *Capn9* ^−/−^ mouse in lysis buffer (10 mM Tris/Cl (pH 7.5), 5 mM EDTA, 150 mM NaCl, 0.5% NP-40, 0.2 mM PMSF, 5 µM pepstatin A, and 25 µM leupeptin) was prepared and immunoprecipitated with or without an anti-calpain 8 antibody, anti-calpain 9 (V-18) antibody, or antigen absorbed-anti-calpain 9 (V-18) antibody for 16 hours at 4°C, followed by the addition of protein G. After the bound protein G was washed five times with the lysis buffer, the immunoprecipitated proteins were eluted with 0.2 M Glycine (pH 2.7) and mixed with SDS sample buffer. For the incubation assay, the gastric mucosal homogenate from a WT, *Capn8* ^−/−^, or *Capn8^CS/CS^* mouse was prepared in TED buffer (10 mM Tris/Cl (pH 7.5), 1 mM EDTA, 1 mM dithiothreitol) containing 0.2 mM PMSF and 5 µM pepstatin A. The homogenate (20 µg) was incubated at 30°C for 30 min in the absence or presence of 5 mM Ca^2+^ with or without 6 µM recombinant human calpastatin domain 1 fragment or 80 µM E64c. The reaction was stopped by adding SDS sample buffer. The protein samples were subjected to Western blot analysis with the appropriate antibodies. For the gel filtration analysis, the gastric mucosal homogenate from a WT, *Capn8* ^−/−^, or *Capn9^−/−^* mouse was prepared in TED buffer containing 0.2 mM PMSF, 5 µM pepstatin A, and 25 µM leupeptin. The homogenate (1 mg) was applied to a Superdex 200 10/30 column (GE Healthcare) equilibrated with elution buffer (150 mM NaCl in TED buffer). COS7 cells were transfected by electroporation, as described previously [13].

### RT–PCR

For RT-PCR analysis, the total RNAs were extracted from the gastric mucosa of WT, *Capn8* ^+/−^, *Capn8* ^−/−^, *Capn9* ^+/−^, and *Capn9* ^−/−^ mice using a TRIzol reagent (Invitrogen), reverse-transcribed to make the first-strand cDNA, using a First Strand cDNA Synthesis kit (Amersham Bioscience) according to the manufacturer's instructions, and subjected to PCR using ExTaq (TaKaRa). Mouse β-actin was used as an internal control. The primers used are listed in Table 2.

### Statistical analysis

Differences between two mean values were analyzed by Student's t-test, and *P* values<0.05 were defined as statistically significant.
